# Simultaneous Real-Time Monitoring of Oxygen Consumption and Hydrogen Peroxide Production in Cells Using Our Newly Developed Chip-Type Biosensor Device

**DOI:** 10.3389/fphys.2016.00109

**Published:** 2016-03-29

**Authors:** Ankush Prasad, Hiroyuki Kikuchi, Kumi Y. Inoue, Makoto Suzuki, Yamato Sugiura, Tomoya Sugai, Amano Tomonori, Mika Tada, Masaki Kobayashi, Tomokazu Matsue, Shigenobu Kasai

**Affiliations:** ^1^Biomedical Engineering Research Center, Tohoku Institute of TechnologySendai, Japan; ^2^Graduate Department of Environmental Information Engineering, Tohoku Institute of TechnologySendai, Japan; ^3^Graduate School of Environmental Studies, School of Engineering, Advanced Institute for Materials Research, Tohoku UniversitySendai, Japan; ^4^Center for General Education, Tohoku Institute of TechnologySendai, Japan; ^5^Graduate Department of Electronics, Tohoku Institute of TechnologySendai, Japan

**Keywords:** biosensors, hydrogen peroxide, reactive oxygen species, catalytic amperometric biosensor, THP-1 cells, chip-type device, oxidative stress

## Abstract

All living organisms bear its defense mechanism. Immune cells during invasion by foreign body undergoes phagocytosis during which monocyte and neutrophil produces reactive oxygen species (ROS). The ROS generated in animal cells are known to be involved in several diseases and ailments, when generated in excess. Therefore, if the ROS generated in cells can be measured and analyzed precisely, it can be employed in immune function evaluation and disease detection. The aim of the current study is to introduce our newly developed chip-type biosensor device with high specificity and sensitivity. It comprises of counter electrode and working electrodes I and II. The counter electrode is a platinum plate while the working electrodes I and II are platinum microelectrode and osmium-horseradish peroxidase modified gold electrode, respectively which acts as oxygen and hydrogen peroxide (H_2_O_2_) detection sensors. Simultaneous measurement of oxygen consumption and H_2_O_2_ generation were measured in animal cells under the effect of exogenous addition of differentiation inducer, phorbol 12-myristate 13-acetate. The results obtained showed considerable changes in reduction currents in the absence and presence of inducer. Our newly developed chip-type biosensor device is claimed to be a useful tool for real-time monitoring of the respiratory activity and precise detection of H_2_O_2_ in cells. It can thus be widely applied in biomedical research and in clinical trials being an advancement over other H_2_O_2_ detection techniques.

## Introduction

Respiratory burst is the rapid release of reactive oxygen species (ROS) such as superoxide anion radical (O${}_{2}^{•-}$) and hydrogen peroxide (H_2_O_2_) involving a marked increase in oxygen consumption from different kinds of cells (Forman and Torres, 2002; Halliwell and Gutteridge, 2007). Bactericidal action and phagocytosis in immune cells such as neutrophils and monocytes is an important biological defense mechanism and are known to produce ROS (Halliwell and Gutteridge, 2007). Hydrogen peroxide is generated from O${}_{2}^{•-}$ which is a primary ROS generated through the activation of NADPH oxidase (Halliwell and Gutteridge, 2007). Among the ROS generated, H_2_O_2_ is known to be relatively stable and thus function as a signal transducer in the cells (Groeger et al., 2009; Marques et al., 2009).

Reactive oxygen species besides its role in immune response has been known to be associated with various kind of human disease and its excessive production adversely affect the living organisms (Halliwell and Gutteridge, 2007; Auten and Davis, 2009; Brieger et al., 2012). The ROS disproportionately generated in the cell or organisms is known to oxidize nucleic acids, lipids and proteins including important biological components such as enzymes (Gutteridge and Halliwell, 2000; Halliwell and Gutteridge, 2007). Hydrogen peroxide is known to be poorly reactive with minimal capability to oxidize polyunsaturated fatty acids and nucleic acid however it can lead to oxidation of amino acids such as cysteine, tryptophan, tyrosine, histidine and methionine (Hoffmann and Meneghini, 1979; Halliwell et al., 2000). Being less reactive, it however acts as a substrate for highly oxidizing radicals such as hydroxyl (HO^•^) in the presence of metal ions (Barbouti et al., 2001).

Oxidative damage of these biological components are in recent years have been an extensive area of study with respect to its involvement in the onset of various diseases, including diabetes and high blood pressure as well as enhancement of the aging phenomenon and lifestyle-related diseases, such as arteriosclerosis (Bostwick et al., 2000; Oikawa, 2005). Reactive oxygen species detection at the cellular level has always been challenging due to non-availability of techniques for specific detection of different ROS and sensitive for low-level detection at the cellular level. Techniques such as low-level chemiluminescence/ultra-weak photon emission has been used as an indirect tool for deciphering the production of ROS and its role in oxidative radical reaction in various kind of living organism in the past few decades however, specific detection had not been possible (Cadenas et al., 1980; Kobayashi et al., 2009; Prasad and Pospišil, 2011). Direct methods involving electron paramagnetic resonance (EPR) spectroscopy have been used to detect species such as HO^·^, singlet oxygen, carbon centered radicals using spin trapping techniques (Buettner and Mason, 2003; Venkataraman et al., 2004; Rác et al., 2015). In addition, EPR spin trapping being an excellent technique for detection of various ROS, direct detection of H_2_O_2_ however is not possible. During the recent past, fluorescent probe such as amplex red has been used but is known to both lack specificity and limitation with respect to minimum detection limit of H_2_O_2_ (Yadav and Pospíšil, 2012). In addition to this, H_2_O_2_ detecting probes including 3,3 diaminobenzidine (DAB), amplex red (AR), amplex ultra red (AUR) and a europium-tetracycline complex (Eu3Tc) have been compared. The probes were tested for sensitivity to light, toxicity, subcellular localization and capacity to detect H_2_O_2_ *in-vivo* (Šnyrychová et al., 2009). The authors suggested that, the toxicity caused by the exogenous addition of fluorescent probes cannot be completely excluded and thus should be used with utmost caution to avoid artifacts.

With the employment of electrochemical techniques, quantitative analysis, real-time monitoring and imaging of ROS has been made possible (Ahammad, 2013). Also, it is conceivable to improve the selectivity by the use of modified electrode (Ahammad, 2013; Enomoto et al., 2013). Attempts have been made to detect ROS and reactive nitrogen species at the cellular level (Amatore et al., 2003, 2008a,b). During the respiratory burst, oxygen consumption was measured under the effect of phorbol 12-myristate 13-acetate (PMA) using Platinum (Pt) microelectrode in immune cells with a scanning electrochemical microscope (SECM; Kasai et al., 2009, 2014). In addition, Inoue et al. measured in real-time, the production of H_2_O_2_ in human progenitor myeloid leukemia cells differentiated into neutrophil-like (HL-60) using Os-HRP modified electrode (Inoue et al., 2010). Polymer used for the Os-HRP modified electrode was developed by Vreeke et al. (1992). The enzyme HRP is converted to its oxidized form, which is than reduced at the surface of the electrode at 0.0 V by the transfer of the electron via the mediator/electron donor (Os) to generate the electrochemical signal (Ohara, 1995; Prasad et al., 2015; Scheme I).

**Scheme I:**
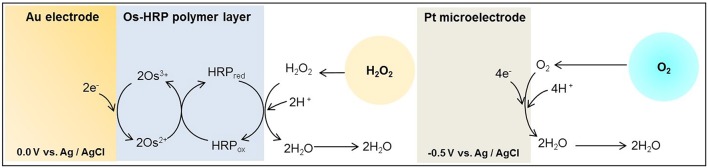
**Schematic illustration of the reaction mechanism of catalytic amperometric method of detection of oxygen consumption using platinum (Pt) microelectrode and H_**2**_O_**2**_ generation using Os-HRP modified gold (Au) electrode**. A Pt plate acts as reference electrode.

Knowing the mechanism and quantitative estimation of ROS formation is crucial and extremely important in biomedical research and clinical applications. In the current study, an electrochemical method for simultaneous detection and quantitative estimation of ROS formation have been demonstrated in THP-1 cells (cell lines of myeloid leukemia patients) during the respiratory burst (Tsuchiya et al., 1980, 1982). THP-1 is a monocyte and are known to differentiate into macrophages under the effect of PMA which acts as a differentiation inducer (Prasad et al., 2015). PMA is a powerful carcinogenic substance, with a similar physiological activity as diacylglycerol (DAG). By directly activating protein kinase C (PKC), it has been known to cause the activation of NADPH oxidase (Castagna et al., 1982; Kikkawa et al., 1983). In the current study, we aimed to design and develop electrochemical sensors that can simultaneously detects in real-time, the consumption of oxygen and production of H_2_O_2_ in animal cells. The real time measurements also reflects oxygen consumption volume and response time during the process of respiratory burst.

## Materials and methods

### Cell culture and reagents

Human monocytic leukemia cell line, THP-1 was purchased from Japanese Collection of Research Bioresources (JCRB) cell bank (Cosmo Bio. Co. Ltd., Tokyo, Japan). The cells were maintained in RPMI 1640 supplemented with 2mM L-glutamine with incubation at 37°C in 5% CO_2_ in humidified atmosphere. The cells were passaged every 3 days.

Glucose, L-glutamine, H_2_O_2_ and PMA were purchased from Wako Pure Chemical Industries, Ltd. (Osaka, Japan) and RPMI 1640 medium and phosphate buffered saline (PBS) were purchased from Sigma Chemical Co. (St. Louis, MO, USA).

### Fabrication of the chip-type electrochemical biosensors and measurement well

Chip-type electrochemical biosensor was fabricated as follows. Glass plate (S1127, Matsunami Glass Ind, Ltd., Japan) was cleaned using the oxygen plasma generator (LTA-101, Yanaco Co., Japan) with conditions as follows: power: 100 W; frequency, 13.6 MHz ; oxygen flow rate, 40 ml/min; time, 3 min. By photolithography, we prepared platinum and gold pattern on a glass substrate. The platinum plate (Pt plate) (1 × 3 mm) was prepared to act as the counter electrode. Platinum (diameter, 20 μm) and gold (diameter, 1 mm) circuit pattern was designed for the detection of oxygen and H_2_O_2_, respectively. For the preparation of Os-HRP modified Au electrode, volume of 0.5 μL Os-HRP polymer solution was immobilized over the electrode surface of the Au electrode and dried overnight at 4°C in the dark (Supplementary data 1). It has been known that, in HRP modified electrodes, electrons from the electrode are relayed to the enzyme through a redox epoxy network to which the enzyme HRP is bound covalently (Vreeke et al., 1992) (Scheme II).

**Scheme II:**
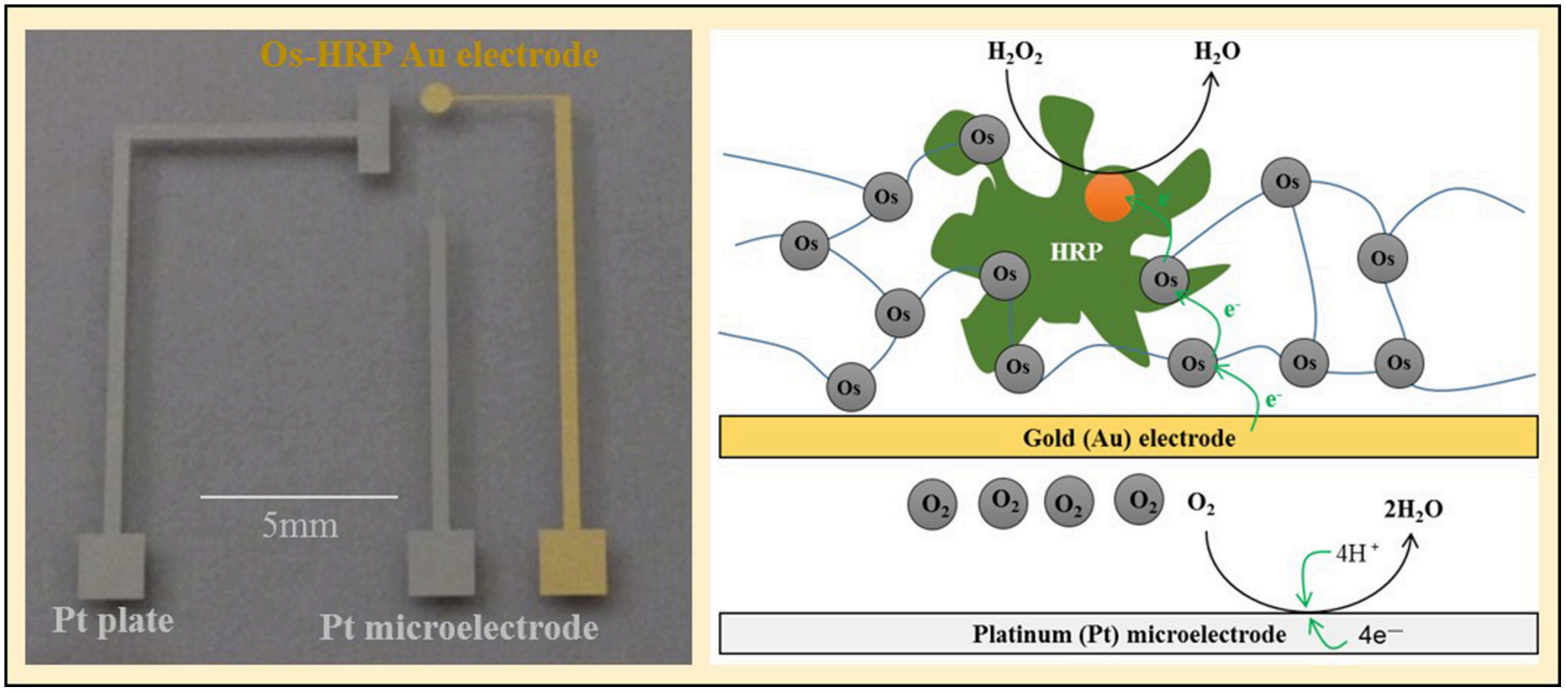
**The chip device showing the Os-HRP modified Au electrode, the Pt microelectrode and Pt plate (left panel) and schematic representation of the mechanism of catalytic amperometry for measurement of oxygen reduction current and reduction current for H_**2**_O_**2**_ using the chip device (right panel)**.

**Figure 1:**
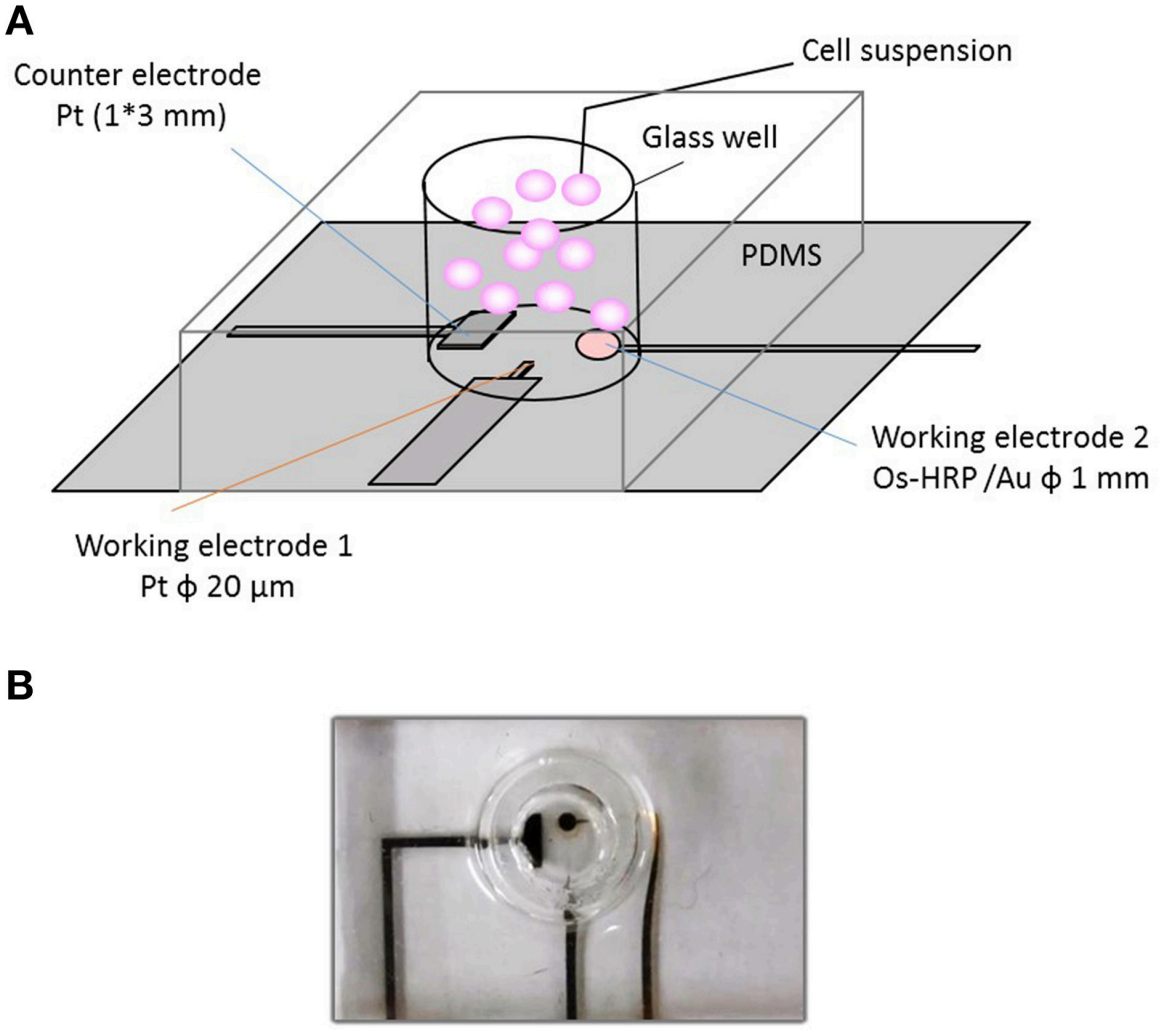
**Experimental setup of catalytic amperometry using newly developed chip-type electrochemical biosensor device**. The setup illustrated shows **(A)**; the schematic representation of the chip-type device showing the counter electrode, CE; working electrode 1, WE1 (Pt microelectrode) and working electrode 2, WE2 (Os-HRP modified Au electrode) **(B)**; photograph of the chip-type device showing the glass well consisting of electrodes.

**Figure 2:**
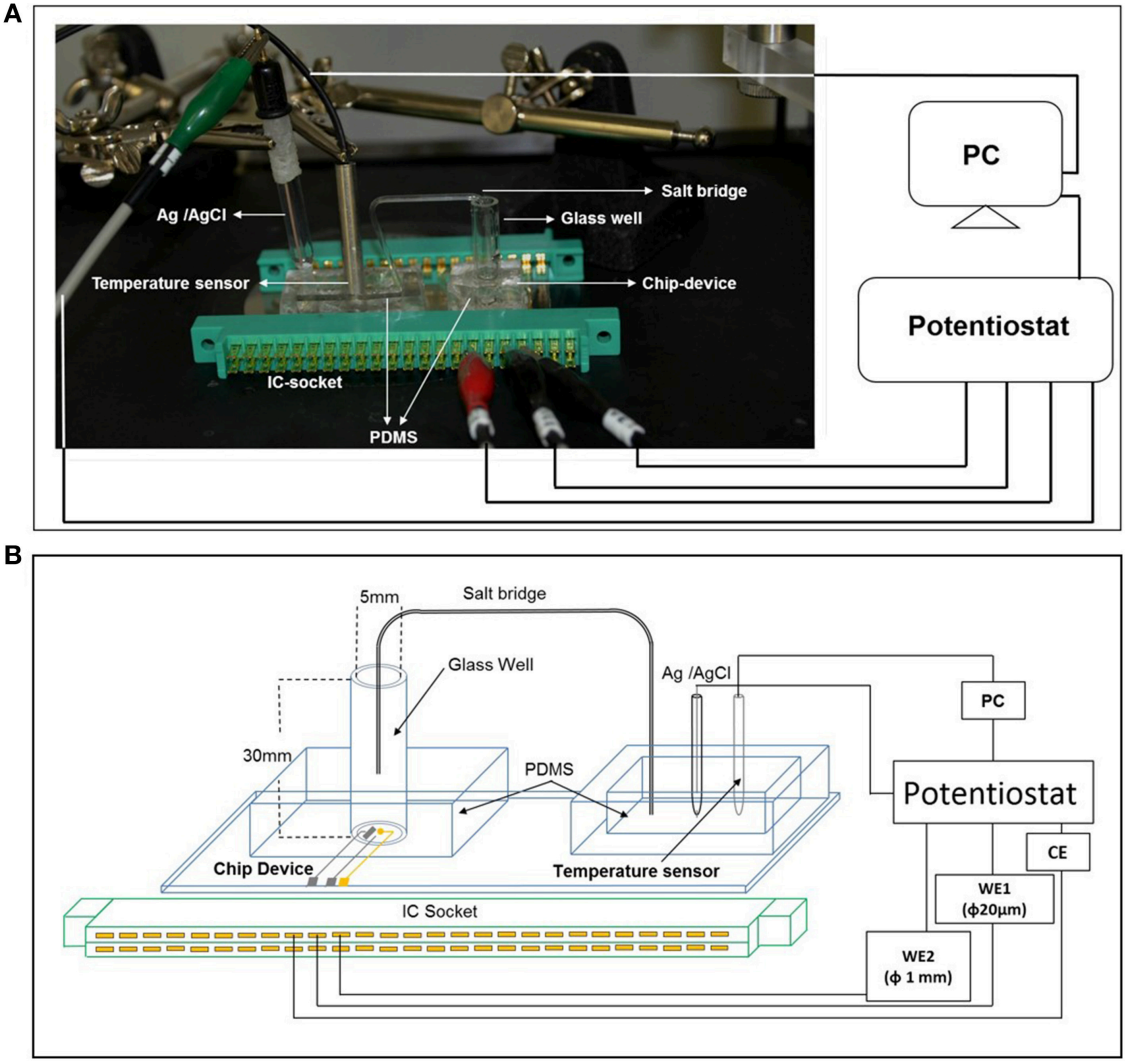
**The photograph showing the arrangement of electrodes (A) and the schematic representation of the experimental setup (B) of catalytic amperometry for measurement of oxygen reduction current and reduction current for H_**2**_O_**2**_**. The setup shows the PDMS prepolymer with a glass tube with an inner and outer diameter of 5 and 7 mm, respectively and height 30 mm containing the chip device; PDMS prepolymer with reference electrode (Ag/AgCl) and the temperature sensor. To avoid any concentration gradient between the 2 wells, a salt bridge was created using a glass tube. Changes in reduction current in WE1 and WE2 were measured using a potentiostat.

For cellular assays, a polydimethylsiloxane (PDMS) prepolymer (SILPOTW/C, DOW Corning Toray, Japan) with a glass tube with an inner and outer diameter of 5 and 7 mm, respectively and height 30 mm (Figure 1A) was poured on an acrylic master plate which was then allowed to solidify with incubation at 90°C for 30 min followed by peeling off from the master plate. Photographs of the chip type device with microelectrodes have been depicted in Figure 1B. A photograph (Figure 2A) and detailed representation of the setup for the electrochemical measurements using chip type device have also been shown (Figure 2B).

### Equipment and methods for electrochemical measurement

**Figure 3:**
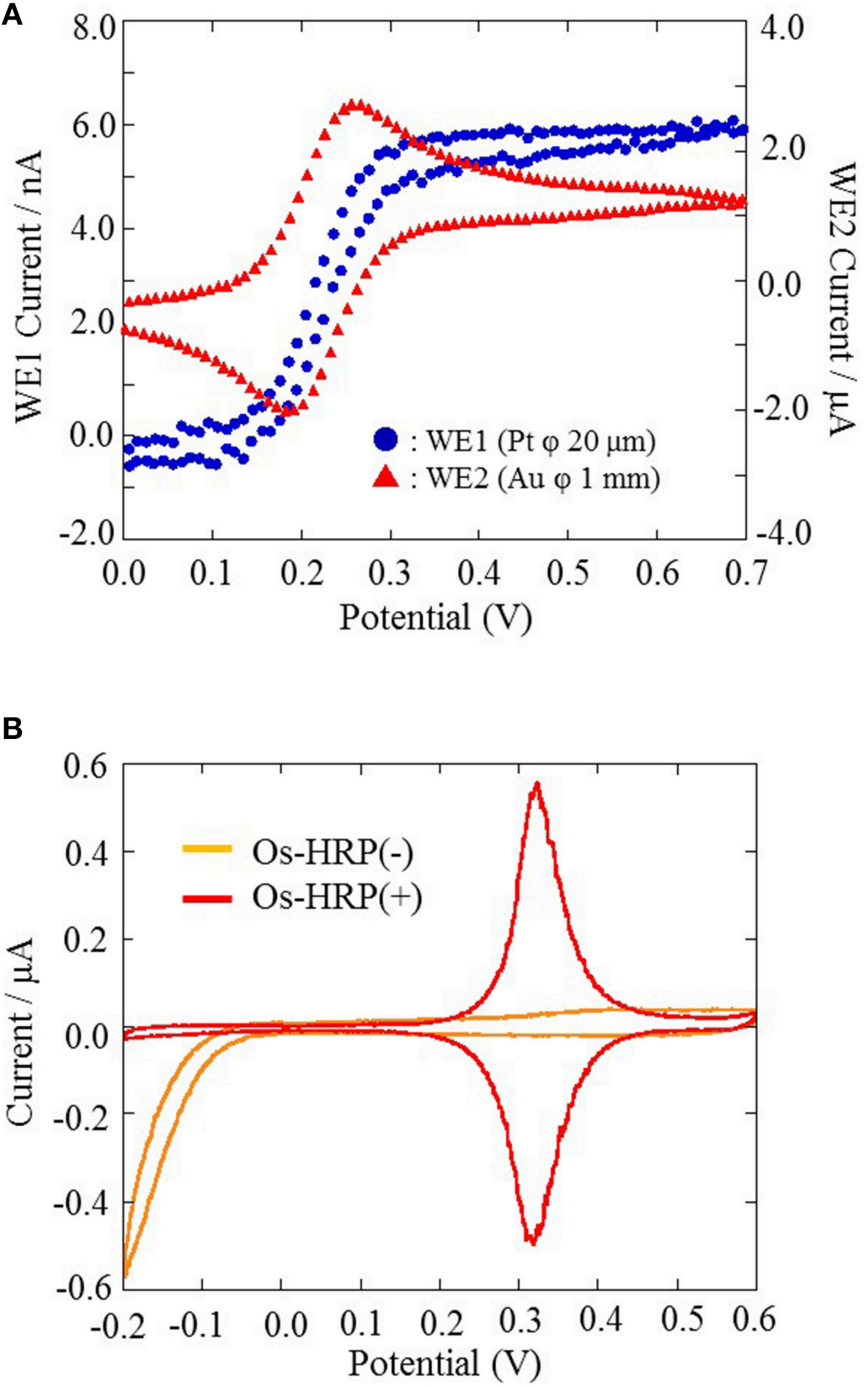
**The characterization of Pt microelectrode and Au electrode performed using cyclic voltammetry at a scan rate of 20 mV/s from 0.0 to +0.7 V at room temperature (A)**. The characterization of Os-HRP modified Au electrode conducted at a scan rate of 20 mV/s from −0.2 to +0.6 V at room temperature **(B)**.

**Figure 4:**
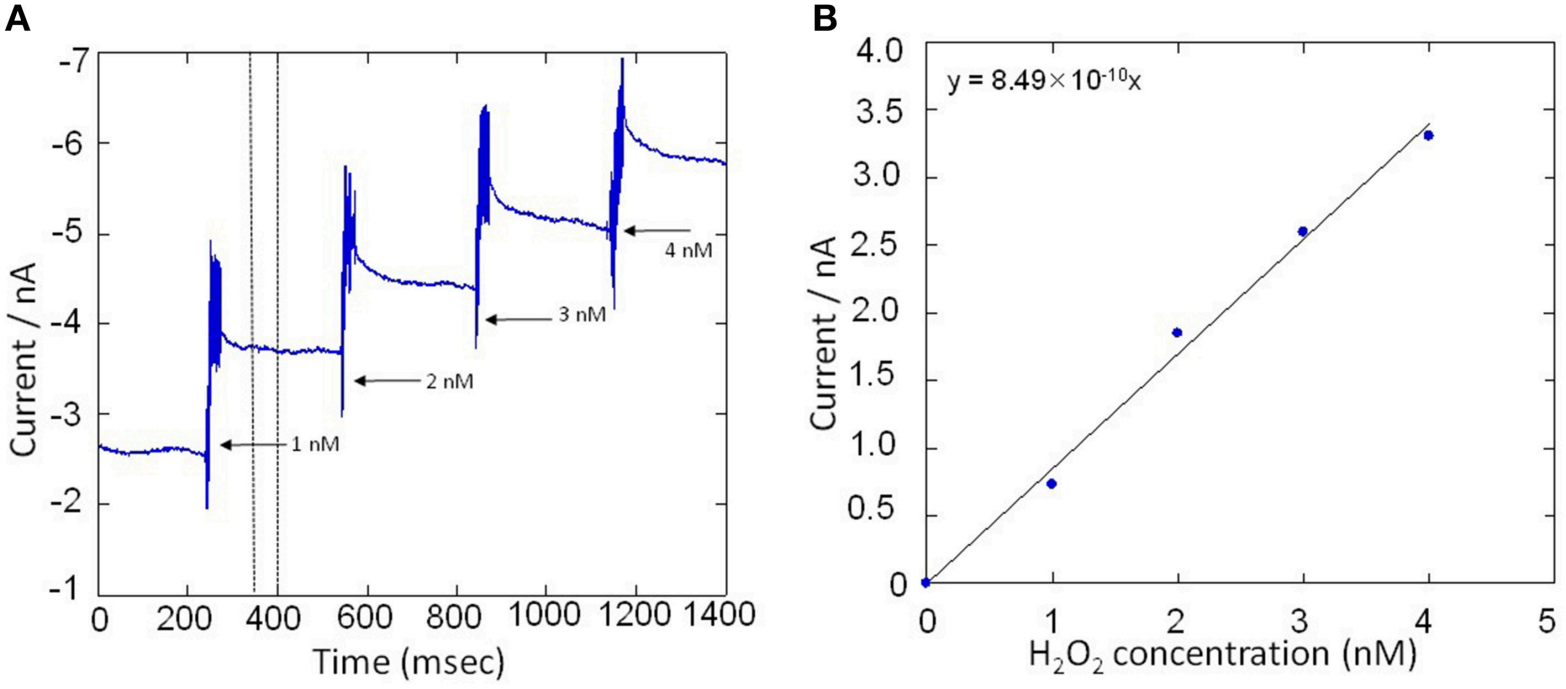
**(A)** Amperometric response of standard H_2_O_2_ solution in PBS in the concentration range of 1–4 nM. Addition of H_2_O_2_ solution was done in real-time under continuous stirring condition and reduction current for H_2_O_2_ was monitored using Os-HPR modified Au electrode. **(B)**, Calibration curve was plotted by taking an average of 50 s of the constant phase (as indicated in **A**, dotted lines) obtained after the addition of standard known concentration of H_2_O_2_.

**Figure 5:**
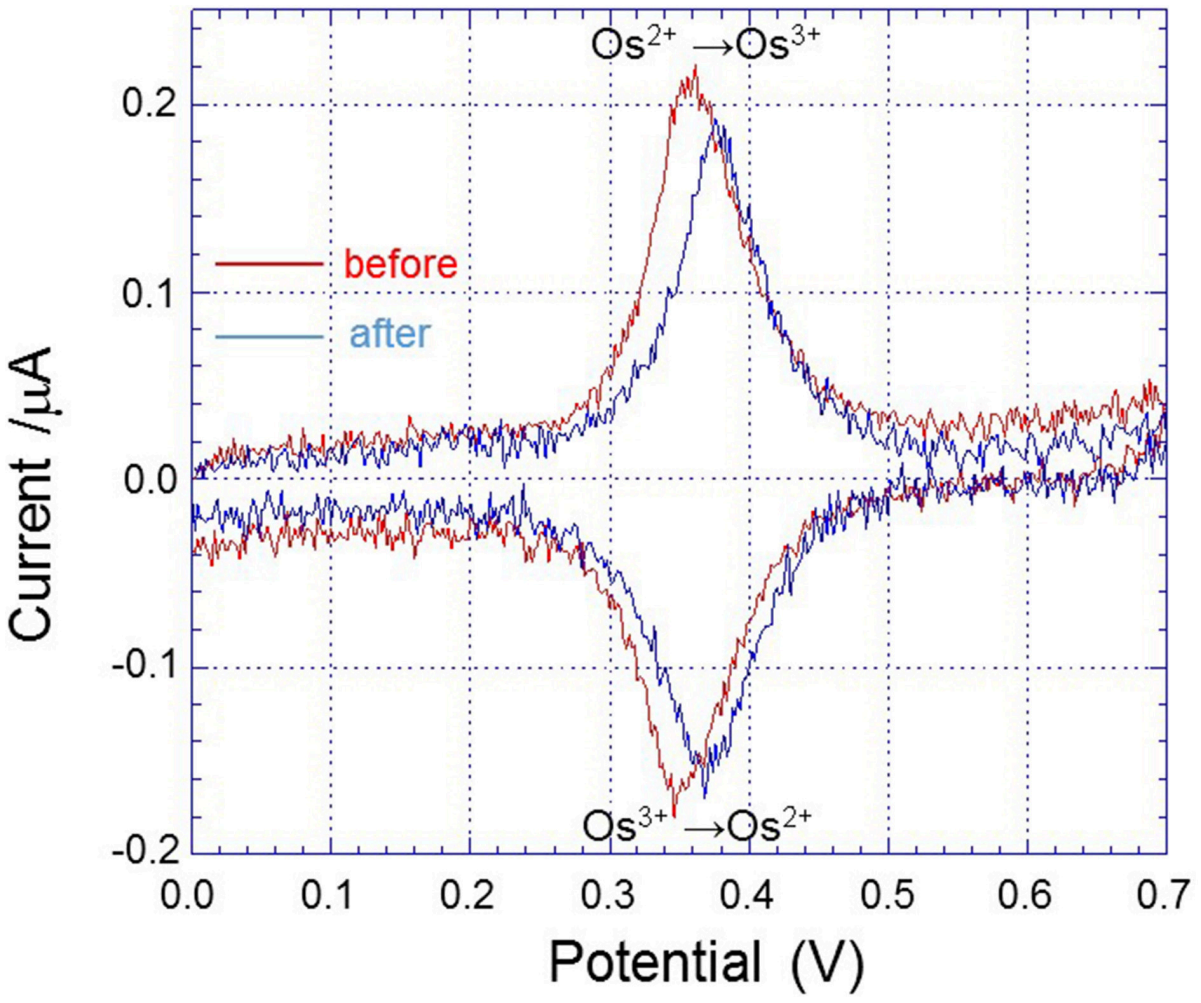
**Cyclic voltammogram of Os-HRP modified Au electrode conducted at a scan rate of 20 mV/s from 0.0 to +0.7 V at room temperature before (red trace) and after 3 h of incubation in 10 μM H_**2**_O_**2**_ (blue trace)**.

All electrochemical measurements were performed using potentiostat (HA1010mM4S; Hokuto denko Co., Ltd., Japan). An Ag/AgCl electrode was used as a reference electrode. For the basic characterization of the Pt microelectrode, Au electrode and Os-HRP modified Au electrode, cyclic voltammetry was conducted at room temperature (Figure 3). Calibration curve of Os-HRP modified Au electrode against standard H_2_O_2_ solution was performed in the concentration range of 1–4 nM at room temperature in PBS solution (Figure 4). Additionally, amperometric response of standard H_2_O_2_ solution in PBS at the final concentration of 0.25 nM H_2_O_2_ (A) and 0.5 nM H_2_O_2_ were also tested (B) **(**Supplementary data 2). To experimentally demonstrate the stability and interaction of Os-HRP on Au electrode, we tested the effect of 10 μM H_2_O_2_ solution by dipping Os-HRP modified Au electrode into it for a span of 3 h. This was followed by measuring the cyclic voltammogram before (red trace) and after 3 h (blue trace). No considerable change in cyclic voltamogram was observed indicating the long term stability of coated Os-HRP on Au electrode (Figure 5).

### Experimental conditions

THP-1 cells were measured in the sensor well at a constant temperature of 30 ± 0.5°C and density of 3.0 × 10^5^ cells/well. Cells were suspended in PBS in presence of glucose at the concentration of 11.4 mM. PMA was added dropwise after 5 min of starting of measurement with concentration of 200 nM and simultaneous reduction currents were measured. Oxygen reduction current was measured at −0.5 V vs. Ag/AgCl and reduction current for H_2_O_2_was measured at 0.0 V vs. Ag/AgCl at room temperature.

## Result and discussion

### Characterization and calibration of electrodes

For the basic characterization of Pt microelectrode and Au electrode, a portion of the electrode were soaked in 10 ml PBS containing 4mM Potassium ferrocyanide [K_4_Fe(CN)_6_] and 0.1 M potassium chloride (KCl) and cyclic voltammetry were performed. Cyclic voltammetry were conducted at a scan rate of 20 mV/s from 0.0 to +0.7 V at room temperature (Figure 3A). For the characterization of the Os-HRP modified Au electrode, cyclic voltammetry was conducted at a scan rate of 20 mV/s from −0.2 to +0.6 V at room temperature. The oxidation and reduction current were obtained at 0.3 V vs. Ag/AgCl (Figure 3B). Based on the data obtained, the surface concentration of Os-HRP on the Au electrode was calculated to be 3.6 × 10^−9^ mol/cm^2^.

Calibration curve of the Os-HRP modified Au electrode was performed to establish the sensitivity and the concentration of H_2_O_2_ formed in THP-1 cells under the effect of PMA. The calibration was done using standard H_2_O_2_ solution in the concentration range of 1–4 nM (Figure 4A). Figure 4B shows a gradual increase in reduction current for H_2_O_2_ upon addition of exogenous standard H_2_O_2_ solution. The change in reduction current for H_2_O_2_ was observed to be approximately 1 nA with each step of 1 nM increase in concentration of H_2_O_2_(Figure 4B). In addition, the response on reduction current for H_2_O_2_ were also measured at concentration below 1 nM (Supplementary data 2). It is thus claimed that the Os-HRP modified Au electrode with the diameter of 1 mm is sufficiently sensitive for H_2_O_2_detection with high precision in concentration range of fractions of nM concentration.

### Simultaneous measurement of oxygen consumption and hydrogen peroxide production

**Figure 6:**
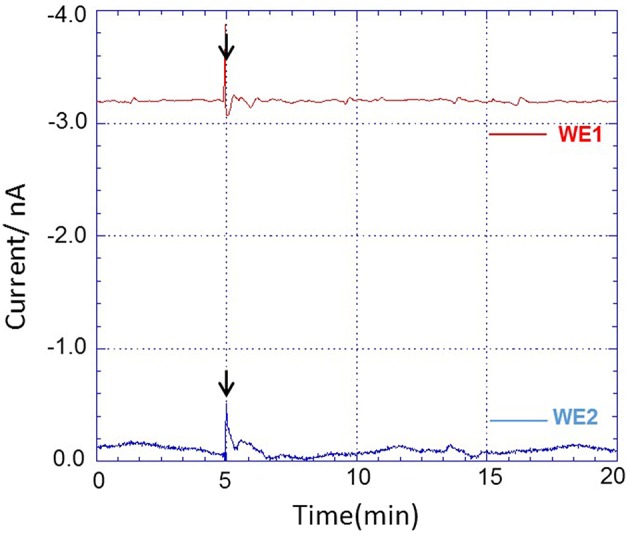
**Amperometric response of addition of PMA dissolved in DMSO (0.001%) in system without THP-1 cells on oxygen reduction current (red trace) measured using WE1 and reduction current for H_**2**_O_**2**_ (blue trace) measured using WE2**. PMA at final concentration of 200 nM was added after 5 min of start of measurement (indicated by arrow).

**Figure 7:**
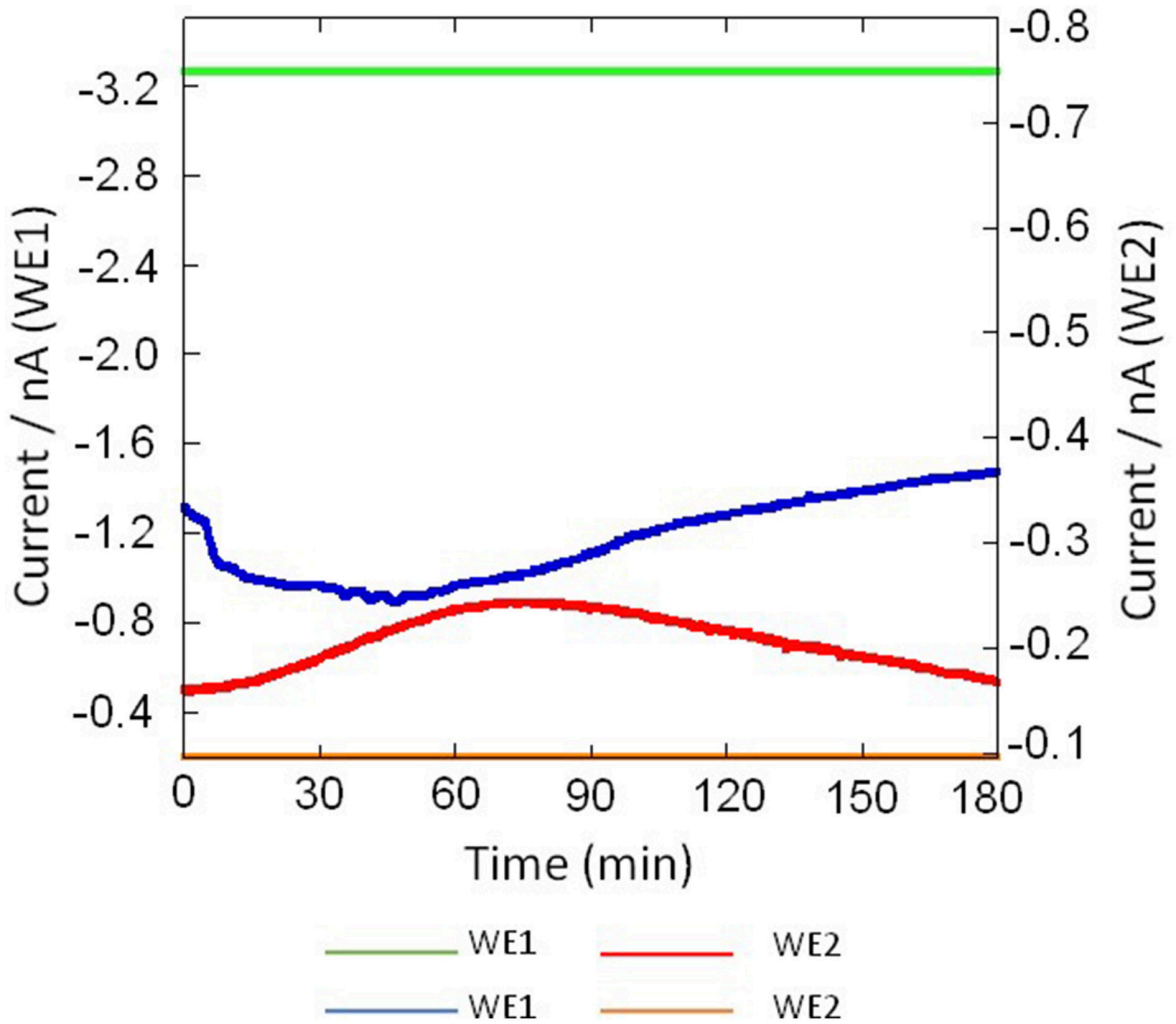
**Real-time monitoring of oxygen reduction current in system containing no THP-1 cell (green trace) and in THP-cell under the effect of 200 nM PMA (blue trace) were measured using Pt microelectrode (WE1) and real-time monitoring of reduction current for H_**2**_O_**2**_ in system containing no THP-1 cell (yellow trace) and during respiratory burst in THP-1 (red trace) using Os-HRP modified Au electrode (WE2) under the effect of 200 nM PMA cells using a chip-type biosensor device**.

Using the chip-type biosensor device comprising of Pt microelectrode and Os-HPR modified Au electrode, the oxygen reduction current and reduction current for H_2_O_2_, respectively were measured at −0.5 V vs. Ag/AgCl and 0.0 V vs. Ag/AgCl. Dimethyl sulfoxide (DMSO) (0.001 %) was used as solvent to solubilize PMA. The amperometric response of PMA (200 nM) in system without THP-1 cells were measured as control to monitor for any changes due to its interference. No significant changes in reduction current for either oxygen or H_2_O_2_ were observed (Figure 6). A variation however, in oxygen reduction current in cell (blue trace) and cell-free system (green trace) was observed because of the fact that the dissolved oxygen concentration is lowered by cell respiration (Figure 7).

**Figure 8:**
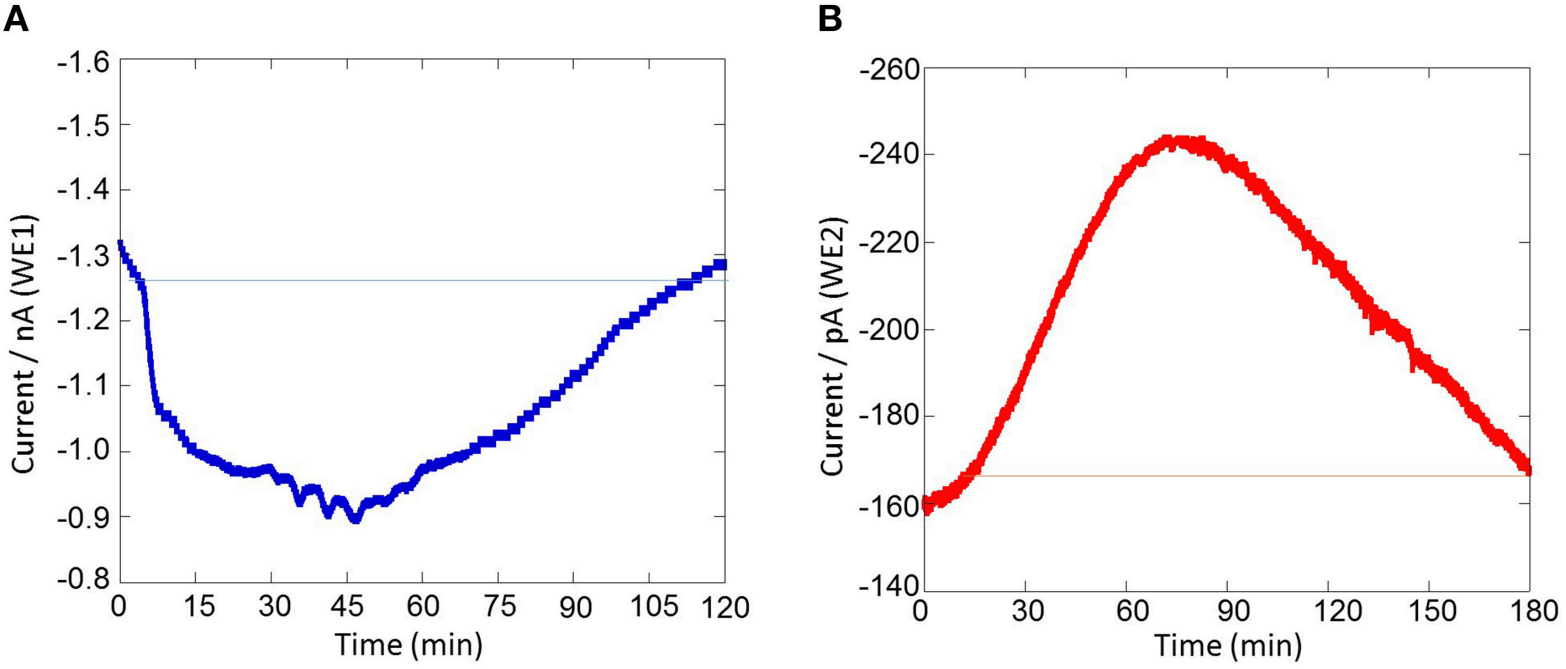
**Real-time monitoring of oxygen reduction current and reduction current for H_**2**_O_**2**_ during respiratory burst in THP-1 under the effect of 200 nM PMA cells using a chip-type biosensor device**. Changes in oxygen reduction current was measured using WE1 **(A)** while changes in reduction current for H_2_O_2_ was measured using WE2 **(B)**.

Figure 7 shows the result of the simultaneous measurement of the oxygen reduction current and reduction current for H_2_O_2_ during the respiratory burst in THP-1 cells with a chip type biosensor device. Oxygen reduction current value was found to decrease immediately after drop wise addition of 200 nM PMA. However, after ~120 min, it showed the same reduction current value as measured before the addition of PMA. This sudden drop is most likely because of the rapid oxygen consumption as a result of respiratory burst in THP-1 cells (Figures 7, 8A). On contrary to immediate change in oxygen reduction current after PMA addition, gradual increase in reduction current for H_2_O_2_ was observed after about 15–20 min of PMA addition. As evident from the kinetics of the reduction current for H_2_O_2_, it can be seen that there occurs continuous production of H_2_O_2_ until ~70 min after PMA addition followed by a gradual decrease (Figures 7, 8B). This indicates that H_2_O_2_ is produced for a longer period of time. From the above observations, it can be concluded that oxygen consumption occurs rapidly as a result of respiratory burst while production of H_2_O_2_ is delayed. In addition, it can be concluded that oxygen consumption and H_2_O_2_ production during respiratory burst are phenomenon which are linked together. The continuous production of H_2_O_2_ during respiratory burst and its longer half-life due to low reactivity can be correlated to involvement of H_2_O_2_ in signaling.

### Sensitivity of the chip-type biosensor device

The results presented shows the real-time monitoring of oxygen consumption and H_2_O_2_ production during respiratory burst in THP-1 cells under the effect of PMA (Figures 7, 8). Before the addition of PMA, an oxygen reduction current value of −1.3 nA was monitored (Figure 8A). It shows the dissolved oxygen concentration in the normal respiratory activity of cells. After the addition of PMA, the oxygen reduction current value was found to rapidly drop by about ~0.3 nA (300 pA) at a concentration of 200 nM PMA (Figure 8A).

**Figure 9:**
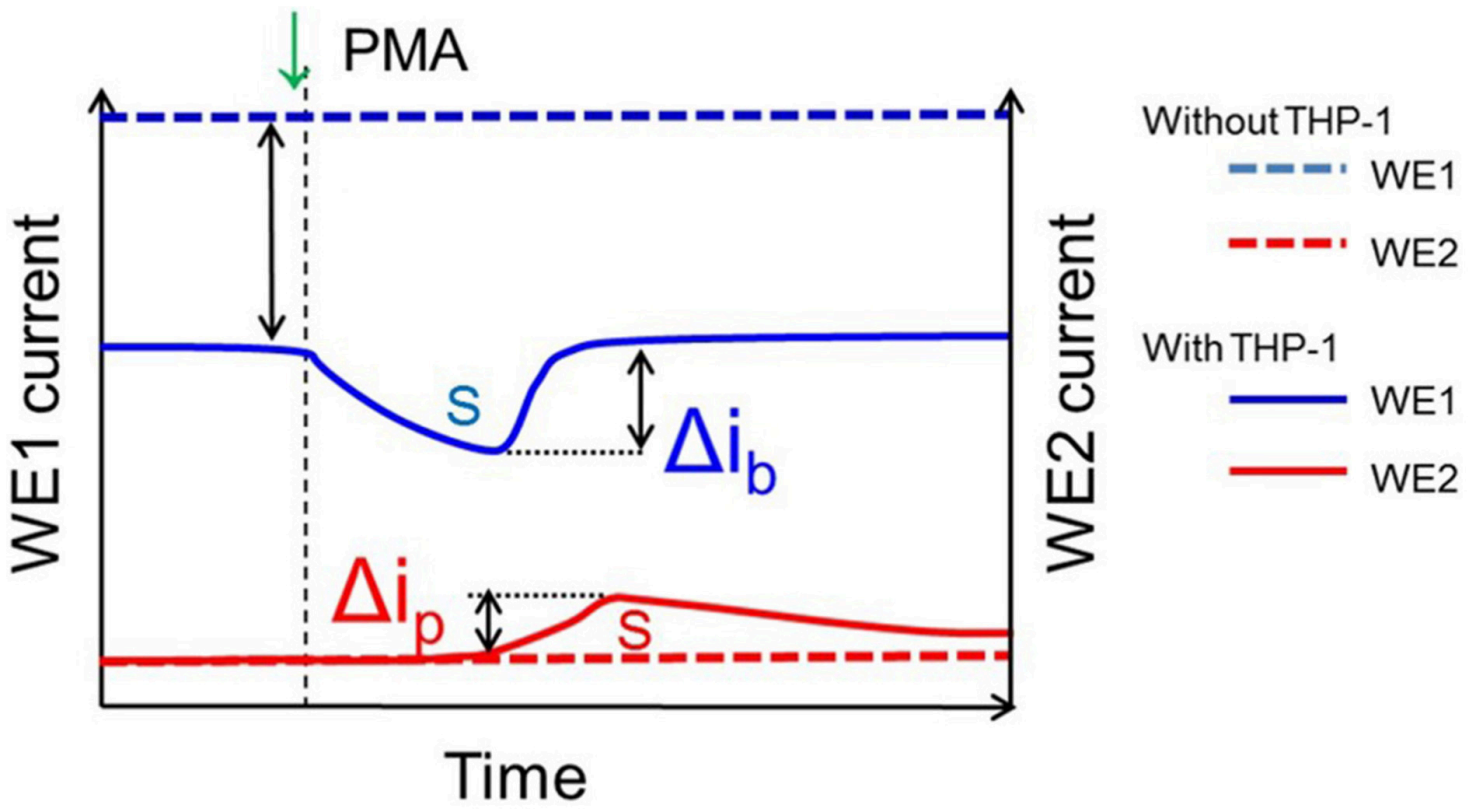
**Details showing parameters utilized for calculation of oxygen consumption and H_**2**_O_**2**_ generation**. The blue line (dotted) shows the bulk oxygen reduction current in system containing no THP-1 cells while the blue line (non-dotted) shows the oxygen reduction current in system containing THP-1 cells; the difference (blue dotted and blue un-dotted represents the change in dissolved oxygen concentration due to cell respiration and Δi_*b*_ represent the change in oxygen reduction current with the exogenous addition of PMA. Similarly, the red line (dotted) shows the reduction current for H_2_O_2_ in system containing no THP-1 cells while the red line (non-dotted) shows reduction current for H_2_O_2_ in system containing THP-1 cells and Δi_*p*_ represent the change in reduction current for H_2_O_2_ with the exogenous addition of PMA. The parameters were considered for calculations of oxygen consumption and generation of H_2_O_2_ as in Table 1.

Similarly, the reduction currents for H_2_O_2_ was measured under the effect of exogenous addition of PMA. With the addition of 200 nM PMA, a net change of 80 pA from −160 pA to −240 pA in reduction current for H_2_O_2_ was observed (Figure 8B). To determine the concentration of H_2_O_2_generated in THP-1 cells, the calibration curve was established for various concentrations obtained using H_2_O_2_ standard solution (Figure 4). Figure 9 depicts the concentration of oxygen consumption and H_2_O_2_ generation in THP-1 cells during respiratory burst. The total oxygen consumption during respiratory burst was found to be equivalent to 297 mM (Table 1A) in the time span of about 110 min recalculated using the standard bulk oxygen concentration as described in Hitoshi and co-workers (Hitoshi et al., 2005) while total concentration of H_2_O_2_ generation in THP-1 cells was recalculated using calibration curve of H_2_O_2_ (Figure 4) and was found equivalent to 494 nM in the time span of about 175 min (Table 1B).

**Table 1:** **Oxygen consumption calculated using standard bulk oxygen of 209 μM and H_**2**_O_**2**_ generation during the respiratory burst calculated using calibration curve (equation y = 8.49 × 10^**−10**^×)**.

**A**			
Δi_b_(pA)	300	S(mM)	297
Δt_b_(min)	110	V[nM/(sec •cell)]	0.15
**B**			
Δi_b_(pA)	80	S(nM)	494
Δt_b_(min)	175	V[fM/(sec •cell)]	0.16

*In (A), the total change in oxygen reduction current was found to be 300 pA (Δi_b_) in the time duration of 110 min (Δt_b_). The total oxygen consumption was found to be 297 mM equivalent to 0.15 nM/sec.cell. In (B), the generation of H_2_O_2_ expressed by change of 80 pA (Δi_p_) in reduction current in the time duration of 175 min (Δt_p_) was recalculated to be equivalent to 494 nM H_2_O_2_. The approximate production of H_2_O_2_ in THP-1 cells was found equivalent to 0.16 fM/sec.cell*.

### Application of the chip-type biosensor device

To validate the potential of chip-type biosensor device for its wide application, we have performed experiments on a different cell line, HL-60 which is a human promyelocytic leukemia cell under the same experimental conditions as in Figures 7, 8. Real-time monitoring of oxygen consumption and H_2_O_2_ generation during respiratory burst in HL-60 cells under the effect of 200 nM PMA at a constant temperature of 30 ± 0.5°C and density of 3.0 × 10^5^ cells/well were measured. Changes in oxygen reduction current was measured using Pt microelectrode, WE1 (red trace) while changes in reduction current for H_2_O_2_ was measured using Os-HRP modified Au electrode, WE2 (blue trace) (Supplementary data 3). Also, real-time monitoring of oxygen reduction current during respiratory burst in HL-60 cells under the effect of 200 nM and 2 μM PMA (*n* = 2) were tested. The time span of oxygen reduction current at different concentration of PMA was observed to vary considerably **(**Supplementary data 4).

**Figure 10:**
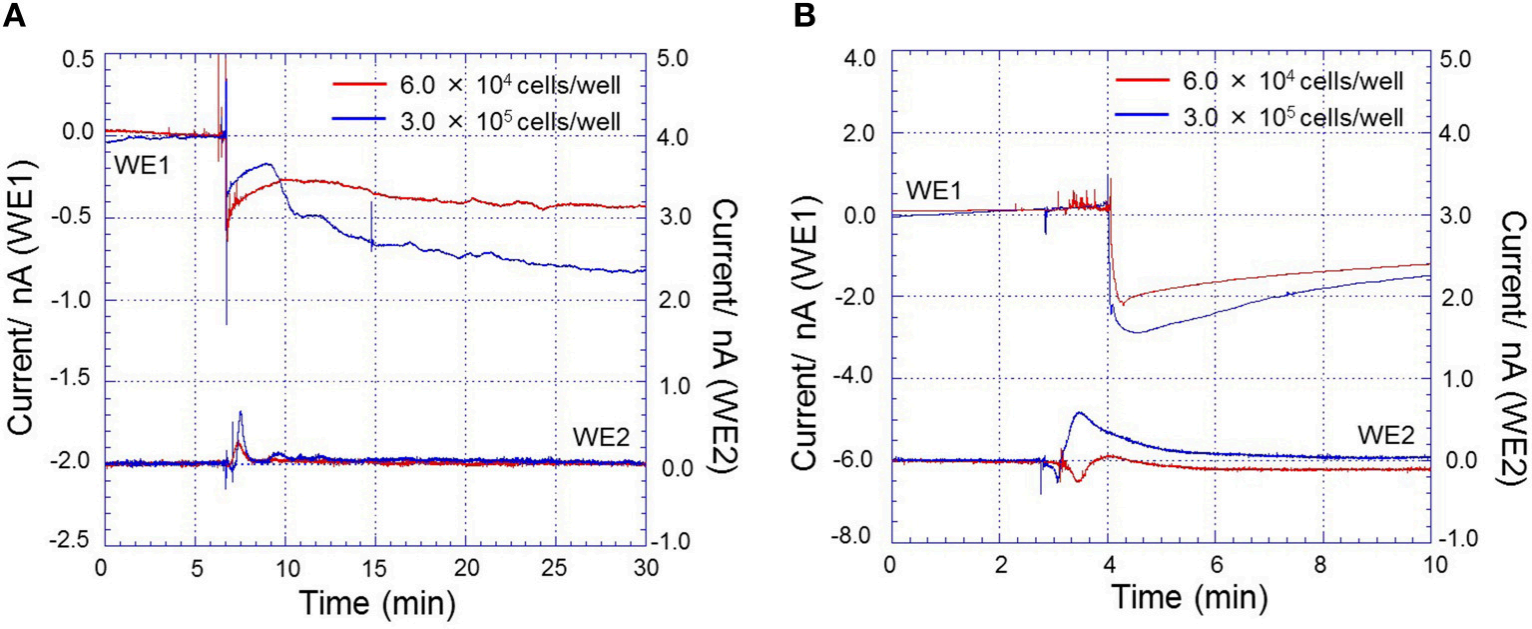
**Real-time monitoring of oxygen reduction current and reduction current for H_**2**_O_**2**_ during respiratory burst in THP-1 cells (A) and HL-60 cells (B) under the effect of 200 nM PMA at a constant temperature of 30 ± 0.5°C and density of 6.0 × 10^**4**^ cells/well (red trace) and 3.0 × 10^**5**^ cells/well (blue trace) using a chip-type biosensor device**. Changes in oxygen reduction current was measured using Pt microelectrode, WE1 while changes in reduction current for H_2_O_2_ was measured using Os-HRP modified Au electrode, WE2.

In addition to this, real-time monitoring of oxygen reduction current and reduction current for H_2_O_2_ during respiratory burst in THP-1 cells (A) and HL-60 cells (B) under the effect of 200 nM PMA at two different cell densities (6.0 × 10^4^ cells/well- red trace and 3.0 × 10^5^ cells/well- blue trace) using a chip-type biosensor device were also tested (Figure 10). Based on these results, it is thus claimed that the chip-type biosensor device can be widely applied in different cell culture with high reproducibility, sensitivity and precision.

## Conclusion

In this study, we have developed a catalytic amperometric chip-type electrochemical biosensor device for simultaneous and real-time monitoring of the respiratory activity and H_2_O_2_ production in animal cells. This device comprises of an Os-HRP modified Au electrode and Pt microelectrode for detection of H_2_O_2_ production and oxygen consumption, respectively. The device is composed of PDMS wells designed to accommodate living cells under controlled conditions. This electrochemical chip-type biosensor device monitored by changes in reduction currents reflecting oxygen and hydrogen peroxide generation is a useful tool for real-time monitoring of the respiratory activity and precise detection of level of oxygen and H_2_O_2_ in cellular systems. It is thus claimed to bear the potential for its application in biomedical research and for paving its way to clinical application being an advancement over other H_2_O_2_ detection techniques.

## Author contributions

HK, TS, MS, AT, and YS carried out the fabrication of the device and the measurements. AP analyzed, interpreted the data and drafted the manuscript. KI, AP, TM, and SK contributed to the conception and design of the work. KI and SK revised it critically for important content. All authors approved the final version of the manuscript.

### Conflict of interest statement

The authors declare that the research was conducted in the absence of any commercial or financial relationships that could be construed as a potential conflict of interest.
