# Supplementary material for: Simultaneous Real-Time Monitoring of Oxygen Consumption and Hydrogen Peroxide Production in Cells Using Our Newly Developed Chip-Type Biosensor Device
Source: Front Physiol. 2016 Mar 29;7:109. doi: 10.3389/fphys.2016.00109 (PMC4810025; doi:10.3389/fphys.2016.00109)
Supplement: Supplementary file 1 [file Image1.PDF]

Supplementary data 1

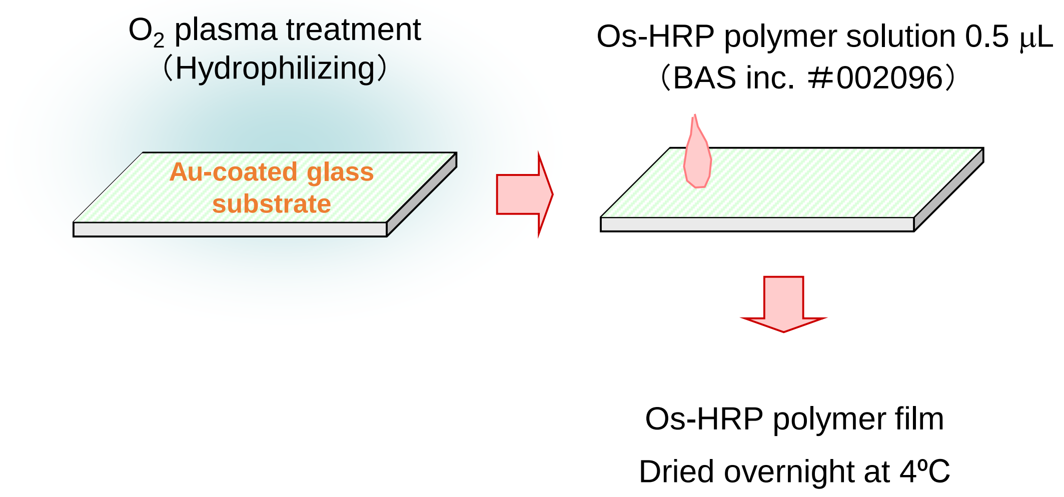

Supplementary data: Steps involved in immobilizing Os-HRP polymer solution on Au electrode
