# Supplementary material for: Simultaneous Real-Time Monitoring of Oxygen Consumption and Hydrogen Peroxide Production in Cells Using Our Newly Developed Chip-Type Biosensor Device
Source: Front Physiol. 2016 Mar 29;7:109. doi: 10.3389/fphys.2016.00109 (PMC4810025; doi:10.3389/fphys.2016.00109)
Supplement: Supplementary file 2 [file Image2.PDF]

Supplementary data 2

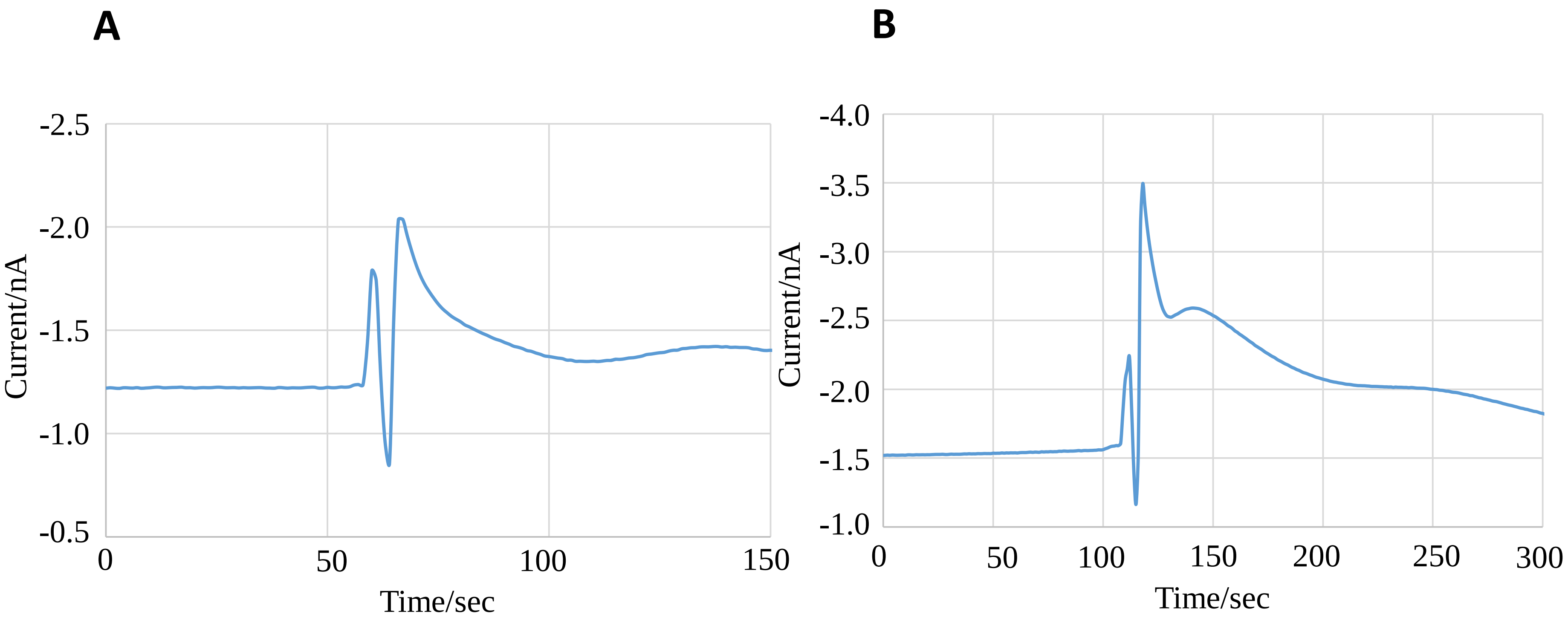

Supplementary data: Amperometric response of standard H_2O_2 solution in PBS at the final concentration of 0.25 nM H_2O_2 (A) and 0.5 nM H_2O_2 (B). Addition of H_2O_2 solution was done in real time under continuous stirring condition and reduction current for H_2O_2 was monitored using Os-HPR modified Au electrode. Calibration time was plotted by taking an average of 50 sec of the constant phase obtained after the addition of standard H_2O_2 between 100 sec-150 sec (A) and 200 sec-250 sec (B).
