# Supplementary material for: Simultaneous Real-Time Monitoring of Oxygen Consumption and Hydrogen Peroxide Production in Cells Using Our Newly Developed Chip-Type Biosensor Device
Source: Front Physiol. 2016 Mar 29;7:109. doi: 10.3389/fphys.2016.00109 (PMC4810025; doi:10.3389/fphys.2016.00109)
Supplement: Supplementary file 3 [file Image3.PDF]

Supplementary data 3

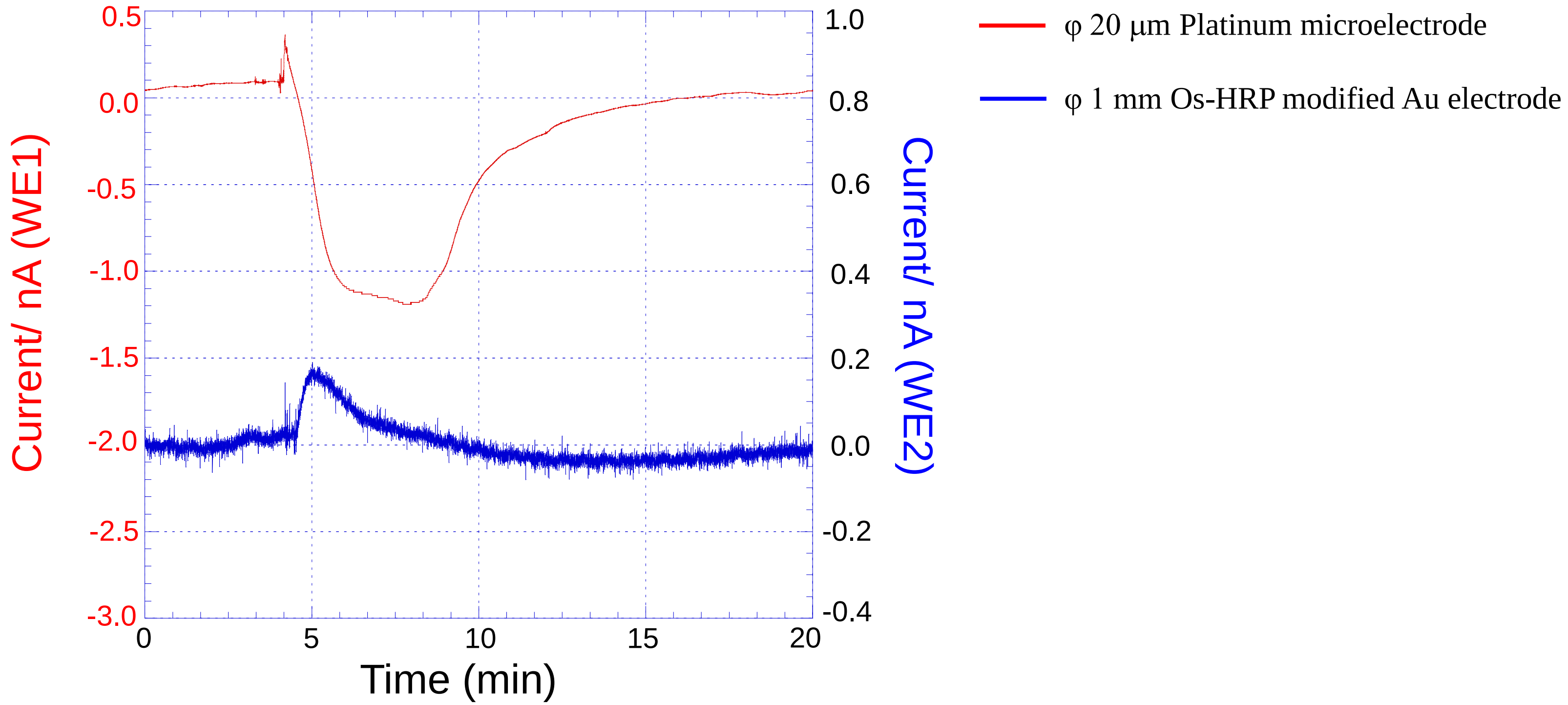

Supplementary data: Real-time monitoring of oxygen reduction current and reduction current for H_2O_2 during respiratory burst in **HL-60 cells** under the effect of 200 nM PMA at a constant temperature of 30 ± 0.5 ° C and density of 3.0 × 10^5 cells/ well using a chip-type biosensor device. Changes in oxygen reduction current was measured using Pt microelectrode, WE1 (red trace) while changes in reduction current for H_2O_2 was measured using Os-HRP Au electrode, WE2 (blue trace).
