# Supplementary material for: Simultaneous Real-Time Monitoring of Oxygen Consumption and Hydrogen Peroxide Production in Cells Using Our Newly Developed Chip-Type Biosensor Device
Source: Front Physiol. 2016 Mar 29;7:109. doi: 10.3389/fphys.2016.00109 (PMC4810025; doi:10.3389/fphys.2016.00109)
Supplement: Supplementary file 4 [file Image4.PDF]

Supplementary data 4

A

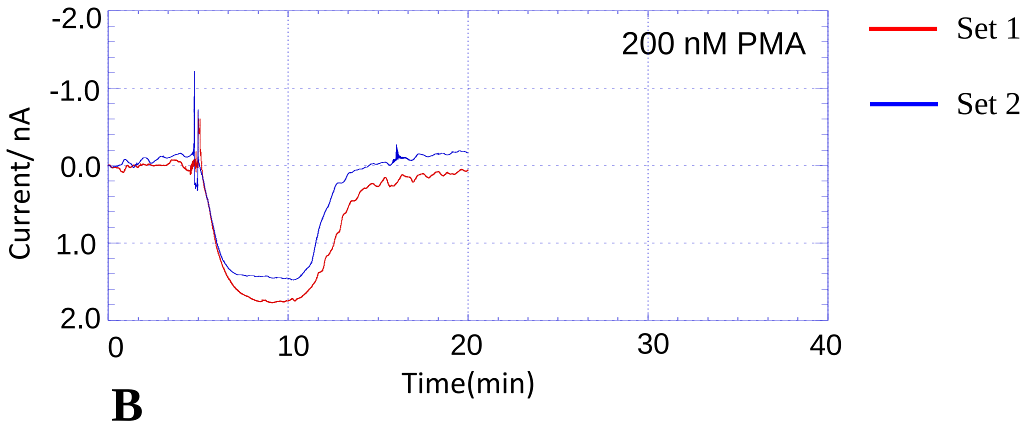

B

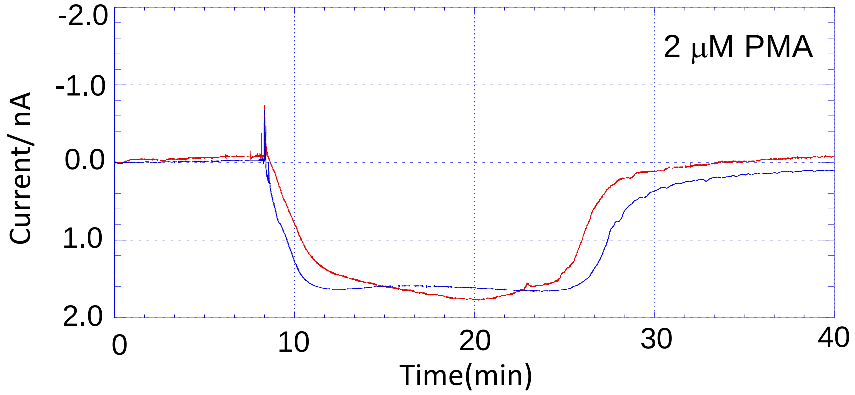

Supplementary data: Real-time monitoring of oxygen reduction current during respiratory burst in HL-60 cells under the effect of 200 nM PMA (A) and 2 μM PMA (B) at a constant temperature of 30 ± 0.5 ° C and density of 3.0 × 10^5 cells/well. Changes in oxygen reduction current was measured using Pt microelectrode, WE1.
